# Supplementary material for: Patterns of Intron Gain and Loss in Fungi
Source: PLoS Biol. 2004 Nov 30;2(12):e422. doi: 10.1371/journal.pbio.0020422 (PMC532390; doi:10.1371/journal.pbio.0020422)
Supplement: Table S1 — Also available at http://genes.mit.edu/NielsenEtAl/. (4.3 MB ZIP). [file pbio.0020422.st001.zip › NielsenEtAl/html/1323.html]

AN4916.1.NCU00258.1.MG00221.1.FG06587.1


```
 CLUSTAL W (1.82) Multiple Sequence Alignments - Introns Inserted


Sequence 1: NCU00258.1	202 aa
Sequence 2: MG00221.1	203 aa
Sequence 3: FG06587.1	203 aa
Sequence 4: AN4916.1	201 aa
Alignment Length: 203 aa
Number Identitical Residues: 147 aa
Alignment Score (without introns) 6136


MG00221.1 	MSSPALNKIAANSPSRQSPSELETNIAQALYDLETNTADLKSALRPLQFVSARE0IEVGH
NCU00258.1	MSAPQLNKIAANSPSRQNPSELETAVAGALYDLESNTADLKAALRPLQFVSARE0IEVGH
FG06587.1 	MSAQALNKIAPNSPSRQNPSELETSIAQALFDLESNTSDLKVALRPLQIVSARE0IEVGH
AN4916.1  	--MAAINKIAHNSPSRQNPSELETAIAGALFDLESNTQDLKATLRPLQFVSARE0VEVGH
          	     :**** ******.****** :* **:***:** *** :*****:***** :****

MG00221.1 	GKKAIVIFVPVPSLQGFHRVQQR2LTRELEKKFSDRHVLILASRRILPRPKRSNRSRNTQ
NCU00258.1	GKKAIVIFVPVPSLQGFHRVQQR2LTRELEKKFSDRHVLIVASRRILPRPKRSSRSRNTL
FG06587.1 	GKKAIVIFVPVPSLQGFHRVQQR2LTRELEKKFSDRHVLILASRRILPRPKRSARSRNNQ
AN4916.1  	GKKAVIIFVPVPLLQAFHKIQQR2LTRELEKKFSDRHVLFVAQRRILPKPKRSVNSRTNQ
          	****::****** **.**::*** ***************::*.*****:**** .**.. 

MG00221.1 	KQKRPRSRTLTAVHDAILTDLVYPVEIVGKRLRTKEDNSKTLKVVLDEKERGGVDYRLDT
NCU00258.1	KQKRPRSRTLTAVHDAILTDLVYPVEIVGKRLRVKEDGSKTLKVILDEKERGSVDYRLDT
FG06587.1 	KQKRPRSRTLTAVHDAILEDLTFPVEIVGKRVRTKEDGSKLLKVILDEKERGGVDYRLDT
AN4916.1  	KQKRPRSRTLTAVHDAILDDLVYPVEIVGKRIRTKEDGSKTLKVILDEKERGGVDHRLDA
          	****************** **.:********:*.***.** ***:*******.**:***:

MG00221.1 	YSEVYRRLTGRSVVFEFPQTGASDY
NCU00258.1	YSAVYRRLTGKNVLFEFPLVSA-EY
FG06587.1 	YSEVYRRLTGRNVNFEFPQSGPADY
AN4916.1  	YGEVYRRLTGRAVVFEFPQGGASEF
          	*. *******: * ****  ..:::
```
